# A comparison of body composition assessment methods in climbers: Which is better?

**DOI:** 10.1371/journal.pone.0224291

**Published:** 2019-11-20

**Authors:** María José Arias Téllez, Fernando Carrasco, Vanesa España Romero, Jorge Inostroza, Alejandro Bustamante, Ignacio Solar Altamirano

**Affiliations:** 1 Department of Nutrition, Faculty of Medicine, University of Chile, Independence, Santiago, Chile; 2 PROFITH “PRO-moting FITness and Health through physical activity” research group, Department of Physical and Sports Education, Sport and Health University Research Institute (iMUDS), Faculty of Sports Science, University of Granada, Granada, Spain; 3 MOVE-IT Research group,Department of Physical Education, Faculty of Education Sciences University of Cádiz, Cádiz, Spain; 4 Biomedical Research and Innovation Institute of Cádiz (INiBICA) Research Unit, Puerta del Mar University Hospital University of Cádiz, Cádiz, Spain; 5 Department of Kinesiology, Faculty of Medicine, University of Chile, Independence, Santiago, Chile; 6 High Performance Center, Pedro de Valdivia, Ñuñoa, Santiago, Chile; University of Mississippi, UNITED STATES

## Abstract

**Objective:**

To compare body composition estimations of field estimation methods: Durnin & Womersley anthropometry (DW-ANT), bioelectrical impedance analysis (BIA) and Deborah-Kerr anthropometry (DK-ANT) against dual-energy X-ray absorptiometry (DXA) in a male Chilean sport climbing sample.

**Methods:**

30 adult male climbers of different performance levels participated in the study. A DXA scan (Lunar Prodigy^®^) was used to determine fat mass, lean mass and total bone mineral content (BMC). Total muscle mass (MM, kg) was estimated through a validated prediction model. DW-ANT and BIA (“non-athletes” and “athletes” equations) were used to determinate fat mass percentage (FM %), while DK-ANT was utilized to estimate MM and BMC.

**Results:**

A significant (p<0.01) inter-method difference was observed for all methods analyzed. When compared to DXA, DW-ANT and BIA underestimated FM% and DK-ANT overestimated MM and BMC (All p<0.01). The inter-method differences was lower for DW-ANT.

**Discussion:**

We found that body composition estimation in climbers is highly method dependent. If DXA is not available, DW-ANT for FM% has a lower bias of estimation than BIA in young male Chilean climbers. For MM and BMC, further studies are needed to compare and estimate the DK-ANT bias level. For both methods, correction equations for specific climbing population should be considered.

## Introduction

**Fig 1:**
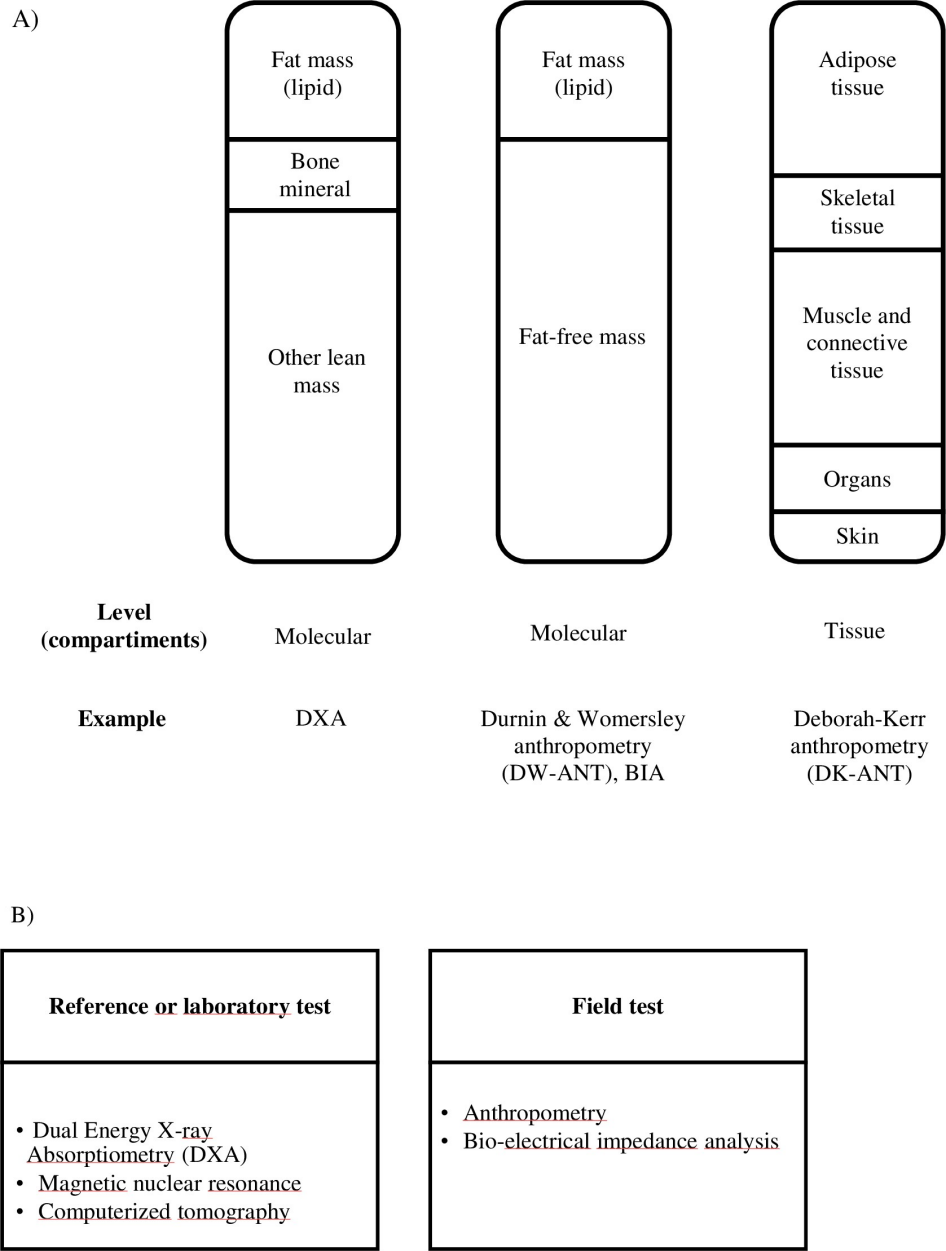
Types of levels of human body composition. A) According to level of analysis B) According to degree of accuracy of referent or field tests.

Body composition assessment is a crucial component in athlete monitoring, not only as a performance-related variable, but also as a training and dietary intervention follow-up, morphological optimization advisory, and health and weight control–especially in weight-sensitive sports [1]. The methods for its evaluation can be classified, according to analysis techniques, in: i) reference tests of molecular level (dual-energy X-ray absorptiometry [DXA], computerized tomography, magnetic resonance imaging) ii) field tests: a) with molecular level: anthropometry (example: Durnin & Womersley anthropometry) or bioelectrical impedance analysis (BIA)[1], and b) tissue anthropometry (example: Deborah-Kerr anthropometry [2] (Fig 1).

Body composition estimation with molecular anthropometry and BIA equations have been developed and validated from general population samples. Thus, the techniques could be less valid on physically active or high performance individuals [1,3]. Additionally, although the anthropometry method at tissue level developed by Kerr [4] has been described in athlete populations [5–7], there is no evidence of its validation against reference methods for different sport disciplines.

With the climbers’ population, there is a topic of relevance because low fat mass percentage (FM%) has been widely described as a determinant of performance [8–14]. As far as it is known, the only study assessing the concordance of “on field techniques” and a “reference technique” was conducted by España-Romero et al (8). They compared 17 anthropometry equations for FM% estimation against DXA. The authors reported that the Durnin & Womersley [15] body density equation, using both Siri’s or Brozek FM% equations [16], did not have significant inter-method difference in female and male climbers.

Anthropometry and BIA are widely used methods because they are easy to apply and inexpensive. Nevertheless, no studies analyzing the molecular and tissue level methods, for the estimation of fat mass (FM) and muscle mass (MM), respectively, at the same time in a specific sport modality has been found. Therefore, the aim of the present study was to compare body composition estimations of *field* estimation methods (Durnin & Womersley anthropometry, bioelectrical impedance analysis and Deborah-Kerr anthropometry) against dual-energy X-ray absorptiometry in a male Chilean sport climbing sample.

## Materials and methods

### Research design and participants

This cross-sectional study included a convenience sample of 30 male climbers (26.1 ± 4.9 years old). Inclusion criteria were being >18 years old, having climbing experience >3 months, free of disease or musculoskeletal injury. The climbers’ levels of performance were assessed trough self-report using the International Rock Climbing Research Association’s (IRCRA) recommendations [17,18]. A comprehensive verbal description of the nature and purpose of the study, as well as the experimental risks were given to the participants. Written informed consent was obtained before participation. The study protocol was approved by the Faculty of Medicine, University of Chile (n° 217–2016) Ethics Committee and met the requirements of the Declaration of Helsinki and the ethical standards in sports and exercise research [19].

### Classic anthropometry measurements

Weight (kg) and height (m) were measured using a calibrated digital scale SECA (model gmbh & co. kg.Hammer Steindamm 3–25, 22089 Hamburg Germany), and a stadiometer brand SECA (model 220), respectively. BMI (kg/m^2^) was calculated.

### Body composition assessment

Each set of individual dual-energy X-ray absorptiometry (DXA) plus BIA measurements were performed in the same day by the same operator, under at least four hours of fasting conditions and without exercise the previous 24 hours to ensure adequate hydration conditions.

- DXA: A Lunar Prodigy Advance equipment (General Electric Systems, Madison, WI), was used to determine FM (kg, and percentage), lean mass (LM, kg), and total bone mineral content (BMC, kg) DXA device was calibrated with phantoms before each set of measurements. In our laboratory, the variability coefficient was 0.01, 0.40 and 2.90% for FM, LM and BMC, respectively. Total MM (kg) was estimated through a predictive model validated by Kim et al. (21) against magnetic resonance imaging. MM (kg) = (appendicular lean mass (kg) x 1.19) - 1.01.
- Bioelectrical impedance analysis (BIA): A tetrapolar, four frequency BODYSTAT® QuadScan 4000 device was used to determine FM (kg, and percentage) (Isle of Man, IM99 1DQ, BRITISH ISLES / Software Version 3.13). With the subject supine, and at least 30 minutes of previous rest, gel electrodes were placed on the right hand, distally 1 cm. on the knuckles, and proximal, immediately on the distal end of the ulna, and on the right foot, in the middle of metatarsus (distal electrode) and in the middle area of the line that joins the lateral and medial malleoli (proximal electrode). One measurement and two predictive equations provided by the manufacturer (“non-athletes” and “athletes”) were used to estimate FM%. The intra-individual variability coefficient of BIA measures was 3.0% for FM%, in our laboratory.
- Durnin & Womersley anthropometry (DW-ANT): A Lange skinfold caliper was used to make two skinfold thickness measurements at 4 anatomical sites according ISAK protocol[20]. The same trained technician performed the measurements, considering a technical measurement error (TME) of 7.5% for intra-evaluator and 10% inter-evaluator [21]. The right biceps, triceps, subscapular and suprailiac sites were measured. The average of the two measurements was used for the analyses. Body density was calculated by Durnin & Womersley equations [15] and converted into FM % via Siri’s equation [16].
- Deborah Kerr anthropometry equations (DK-ANT)[4]: According to ISAK protocol [20], 10 body perimeters (cm), 6 body diameters (mm), 9 lengths and segments (cm) and 8 skinfolds (mm) were measured using an inextensible metallic tape, anthropometer and caliper. The skinfolds measured were (at the right side): biceps, triceps, subscapular, supra spinal, iliac crest, abdominal, frontal thigh and calf. For intra-evaluator, a TME of 7.5% for skinfolds and 1.5% for other measurements were considered. For inter-evaluator, the TME considered wereETM of 10% and 2% respectively, were tolerated [21]. The average of the two measurements was used for the analyses Muscle mass (MM, kg) and BMC (kg) were estimated using AntropoS2 software (Holway, 2000).

### Statistical analysis

Descriptive statistics were performed to examine the characteristics of the study participants. Agreement between DXA and composition assessment methods was determined using Bland-Altman plots (Bland & Altman, 1986). Validity and lack of agreement between DXA and the equations was assessed by calculating the bias–that is, the inter-method differences and standard deviation (SD) of the differences. The 95% confidence intervals for the bias and the 95% limit of agreements (bias ± 1.96 sd) were also calculated. Differences between methods (equation vs. DXA) were analyzed by paired t-tests. Heteroscedasticity was examined by the Pitman test to determine whether the absolute inter-method difference was associated with the magnitude of the measurement (i.e. inter-methods mean). A significant association (*P*<0.05) would confirm heteroscedasticity. Statistical analyses were performed using STATA software version 14.0.

## Results

Taking into consideration the IRCRA ability grouping statement, 9 novice climbers, 11 advanced climbers and 10 elite climbers composed the sample. The main characteristics of the participants are presented in Table 1.

**Table 1:** Characteristics and body composition of the participants.

	All (n = 30)	Range
Age (y.)	26.2 ± 4.90	18.0–36.0
Weight (kg)	66.1 ± 6.40	55.1–79.5
Height (m)	1.73 ± 0.10	1.61–1.88
BMI (kg/m^2^)	22.1 ± 1.50	20.0–25.5
Total fat mass DXA (kg)	10.1 ± 2.20	6.15–15.0
Total fat mass DXA (%)	15.0 ± 2.80	10.8–21.2
Total lean mass DXA (kg)	53.9 ± 5.10	44.6–62.8
Total estimated muscle mass DXA (kg)	29.3 ± 3.30	23.6–37.4
Total bone mineral content DXA (kg)	2.90 ± 0.40	2.19–3.60

Values are mean ± standard deviation and range. DXA: dual energy X-ray absorptiometry.

Estimated FM%, MM and BMC estimation and the inter-method differences for BIA, DW-ANT, and DK-ANT are shown in Tables 2 and 3. A significant (*P*<0.01) inter-method difference was observed for all methods analyzed. DW-ANT and BIA underestimated FM%, and DK-ANT overestimated MM and BMC when they were compared to DXA. Specifically, the range of 95% limit of agreement was between 5.45 and 11.10% to calculate FM% (11.10, 8.86 and 5.45% for the BIA athlete equation, BIA non- athlete equation and DW-ANT, respectively), and between 9.95 and 4.04 kg to calculate MM and BMC with DK-ANT method. Further, it was observed heteroscedasticity (increase in the variance with increase in the magnitude) in the BIA athlete equation (P = 0.014), muscle mass (P = 0.031) and bone mineral content (<0.001).

**Table 2:** Inter-methods differences in fat mass estimation: BIA (athlete and non-athlete equations) and Durnin & Womersley anthropometry (DW-ANT) against DXA.

Method	Fat mass (%)	Method–DXA	*95% Limits of agreements*	P*
	Mean ± SD	(95% CI)		
DXA	15.04 ± 2.79	-	-	-
BIA athletes	8.80 ± 1.80	-6.24 (-7.27 to -5.20)	-11.79 to -0.69	0.014
BIA non-athletes	5.87 ± 2.30	-9.17 (-9.99 to -8.34)	-13.60 to -4.74	0.163
DW-ANT	12.30 ± 3.20	-2.73 (-3.39 to -2.06)	-6.30 to 0.85	0.154

*Heterocedasticity by Pitman's Test.

BIA, bioelectrical impedance analysis; DXA, dual-energy X-ray absorptiometry.

**Table 3:** Inter-methods differences in muscle mass and bone mineral content estimation: anthropometry (DK-ANT) against DXA.

Methods	Mean ± SD	Method—DXA	*95% Limits of agreements*	P*
		(95% CI)		
DXA MM (kg)	29.3 ± 3.30	-	-	
DK-ANT MM (kg)	34.5 ± 4.30	5.23 (4.30 to 6.16)	0.25 to 10.20	0.031
DXA BMC (kg)	2.91 ± 0.37	-	-	
DK-ANT BCM (kg)	7.54 ± 1.20	4.62 (4.24 to 4.99)	2.60 to 6.64	<0.001

*Heterocedasticity by Pitman's Test.

DXA, dual-energy X-ray absorptiometry; MM, muscle mass; BCM, bone mineral content.

**Fig 2:**
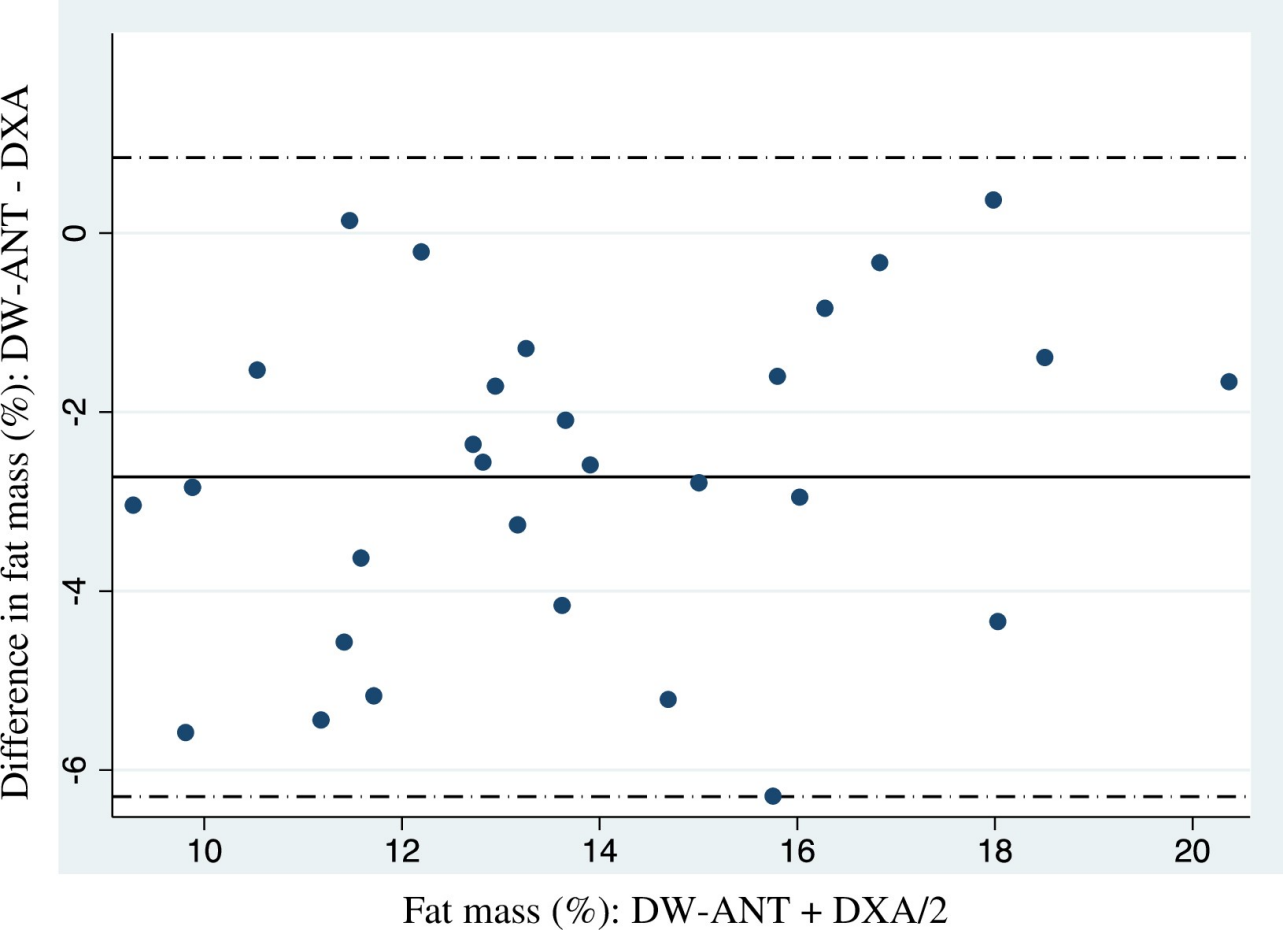
Comparison of predicted percentage of fat mass (FM %) between Durnin & Womersley anthropometry (DW-ANT) and dual-energy X-ray absorptiometry (DXA) in climbers by Bland and Altman plots. The central line represents inter-method differences. Upper and lower broken lines represent the 95% limit of agreement (inter-methods difference ± 1.96 sd of the differences).

**Fig 3:**
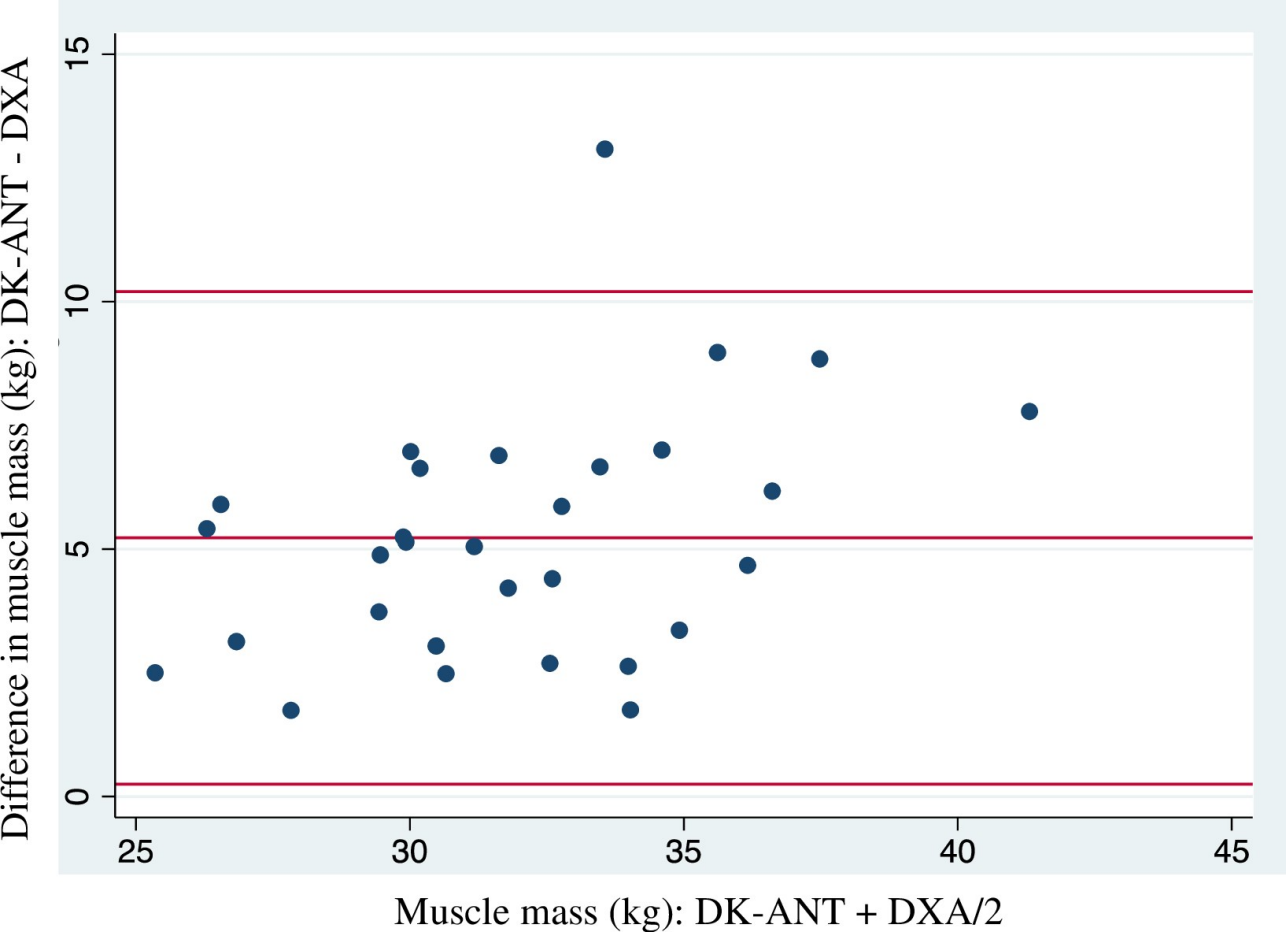
Comparison of predicted muscle mass (kg) between Deborah-Kerr anthropometry (DK-ANT) and dual-energy X-ray absorptiometry (DXA) in climbers by Bland and Altman plots. Central line represents inter-method differences. Upper and lower broken lines represent the 95% limit of agreement (inter-method difference ± 1.96 sd of the differences).

Bland-Altman plot of fat mass with DW-ANT method and MM with DK-ANT method show the smaller inter-method differences are shown in Fig 2 and Fig 3.

## Discussion

The present study compared body composition estimation by three different on-field estimation methods against DXA in a male Chilean sport climbers sample. In general, the results showed that DW-ANT and BIA underestimated FM%, and DK-ANT overestimated MM and BMC when compared with DXA. Moreover, inter-method differences was lower for DW-ANT Therefore, in a field scenario, DW-ANT for FM% has lower bias of estimation than other approaches in young male Chilean climbers. Nevertheless, for MM and BMC, further studies are needed to compare and estimate the DK-ANT bias level.

Our results are in line with the study of España-Romero et al. (10) in rock climbers. They suggested an anthropometry equations, i.e., Durnin & Womersley body density equation using both, Siri’s or Brozek FM% equations [16], as the most accurate to estimate FM% among men elite climbers. Nevertheless, the lack of previous studies for validation of DK-ANT method against reference methods, does not allow performing more comparisons.

Previous studies have compared field method as anthropometry and BIA for other specific athlete populations. Specifically, Lopez-Taylor et al. analysed the accuracy of 31 anthropometry equations to estimate body FM% among professional male soccer players using DXA. Only 12 equations showed non-significant differences with DXA and 4 showed narrower limits of agreement [22]. Lozano et al. found significant differences for FM% estimation using DXA, anthropometry (Slaughter equation), BIA and air displacement plethysmography in young football players, proposing these methods are not interchangeable in this population [23]. Similar results found Zemski et al. and Carvalho et al. when comparing skinfold equations against DXA in professional rugby players [24,25]. The existing formulas tended to underestimate FM% at low levels of adiposity, whilst overestimating it at the higher levels. Other sport disciplines and comparisons can be found in the review of Mazic et al., 2014 [26]. Finally, as far as we know, our FM% estimation -both with on field methods and DXA- are in line with those previously described in climbers -with values between 4.7–15.3% [12,27–29] and 13.3–20.4 [8], respectively.

As expected, when FM% was determined using two predictive equations provided by the BIA manufacturer (non-athletes and athletes), it was observed BIA athletes-equation had a smaller difference that BIA non-athletes in the FM% calculation with respect DXA. Nevertheless, the lack of public access to the equations used for the estimation appear as a limitation for further analysis.

Despite the absence of previous studies that validate the estimation of muscle mass with the DK-ANT method, its low cost and lower technical complexity make it attractive as a method in a field setting. However, the results of this study suggest that, when compared to DEXA, DK-ANT does not provide a reliable estimate of muscle mass, unless appropriate equations are developed to decrease the estimation error.

Although BMC for DK-ANT presents smaller inter-methods differences than MM estimated, the higher heteroscedasticity observed -possibly for lack of validity and/or accuracy of lengths and diameters measurements used and the absence of studies about equations to estimate BMC compared with DXA in elite climbers- suggest the need of further analyses in this topic.

Some strengths of the present study must be highlighted. The use of a reference method for the estimation and comparison of body composition, the comparison of techniques in regard to level of analysis of body composition (level II and IV), the inclusion of different performance level climbers and the register of the athletes under the IRCRA classification and grouping statement, allowing comparison in future studies. On the other hand, we acknowledge that some limitations when interpreting and comparing the results could be: i) the lack of previous studies examining the validation of DK-ANT method against reference methods, especially in the climbing population ii) the absence of female athletes and iii) the lack of knowledge of the equations for the estimation of FM% by BIA.

In **conclusion**, according to our findings, the results of body composition analysis in climbers are highly dependent on the methods used. Taken together, we suggest that in the case when DXA is not available, DW-ANT (Durnin & Womersley and Siri equations) for FM% would have advantages over BIA for body composition estimation in Chilean climbers. It is uncertain if the BIA bias could be less with other devices or other equations. Further studies are needed to compare and estimate the DK-ANT bias level. In addition, for both methods, correction equations for specific climbing population should be considered.

## Supporting information

S1 Data Set (RAR)
